# Negative-pressure wound therapy to treat thoracic empyema with COVID-19-related persistent air leaks: A case report

**DOI:** 10.3389/fmed.2022.970239

**Published:** 2022-08-11

**Authors:** Kensuke Konagaya, Hiroyuki Yamamoto, Tomoki Nishida, Tomotaka Morita, Tomoyuki Suda, Jun Isogai, Hiroyuki Murayama, Hidemitsu Ogino

**Affiliations:** ^1^Department of Surgery, Narita-Tomisato Tokushukai Hospital, Chiba, Japan; ^2^Department of Cardiovascular Medicine, Narita-Tomisato Tokushukai Hospital, Chiba, Japan; ^3^Department of General Thoracic Surgery, Shonan Kamakura General Hospital, Kanagawa, Japan; ^4^Department of Anesthesiology, Narita-Tomisato Tokushukai Hospital, Chiba, Japan; ^5^Department of General Surgery, Shonan Kamakura General Hospital, Kanagawa, Japan; ^6^Department of Radiology, Asahi General Hospital, Asahi, Japan

**Keywords:** COVID-19, pneumothorax, persistent air leaks, empyema, open-window thoracostomy, negative-pressure wound therapy

## Abstract

The novel coronavirus disease (COVID-19) has resulted in a global pandemic. Recently, COVID-19-related pneumothorax has gained attention because of the associated prolonged hospital stay and high mortality. While most cases of pneumothorax respond well to conservative and supportive care, some cases of refractory pneumothorax with persistent air leaks (PALs) do not respond to conventional therapies. There is a lack of evidence-based management strategies to this regard. We describe the case of a 73-year-old man with COVID-19-related acute respiratory distress syndrome (ARDS) who developed delayed tension pneumothorax with PALs caused by alveolopleural fistulas. Despite chest tube drainage, autologous blood pleurodesis, and endoscopic procedures, the PALs could not be closed, and were complicated by thoracic empyema. Subsequent minimally invasive open-window thoracostomy (OWT) with vacuum-assisted closure (VAC) therapy helped successfully control the refractory PALs. Serial chest computed tomography monitoring was useful for the early detection of the pneumothorax and understanding of its temporal relationship with air-filled lung cysts. Our case provides a new perspective to the underlying cause of refractory pneumothorax with PALs, secondary to COVID-19-related ARDS, and underscores the potential of OWT with VAC therapy as a therapeutic alternative in such cases.

## Introduction

Coronavirus disease (COVID-19) caused by severe acute respiratory syndrome coronavirus 2 (SARS-CoV-2) has resulted in a global pandemic. Though it can affect all organs, it has a predilection for the respiratory system, where it easily progresses to acute respiratory distress syndrome (ARDS) in severe cases. While the management of the acute and critical phase of COVID-19 has advanced rapidly, the treatment of its sequelae remains a challenge.

Pneumothorax is defined as the presence of air in the pleural space with subsequent impairment of oxygen supply and ventilation. It is a known sequela of COVID-19. The association between COVID-19 and pneumothorax development has recently gained attention because of the latter’s association with prolonged hospitalization and increased in-hospital mortality (1). Pneumothorax is a common complication strongly associated with barotrauma during invasive mechanical ventilation (IMV) (2). However, COVID-19-related pneumothorax can develop in both spontaneous breathing and mechanical ventilation settings (3). It can also develop irrespective of body weight, pre-existing lung disease, or smoking status, which are well-known risk factors for pneumothorax (3). The exact mechanism underlying COVID-19-related pneumothorax therefore remains poorly understood. SARS-CoV-2 infection complicated by pneumothorax is managed by either general supportive care or removal of air from the pleural space through chest tube thoracostomy. Among such cases, the management of refractory cases with persistent air leaks (PALs) remains challenging, owing to its complex diagnosis and lack of evidence-based treatment strategies.

## Case description

**FIGURE 1:**
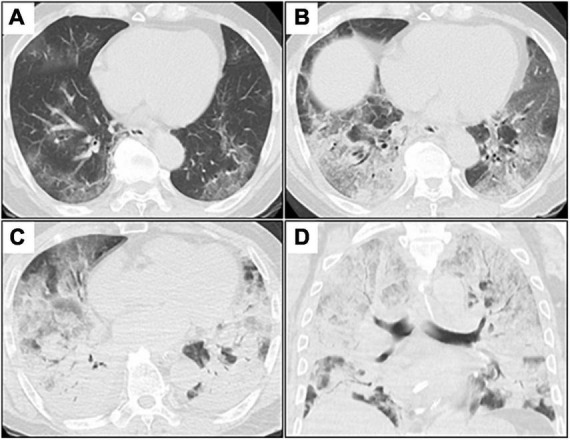
Serial chest CT images after admission. **(A–C)**, axial images; **(D)**, coronal image. **(A)** Initial chest CT shows patchy GGOs in bilateral peripheral lungs. **(B)** Chest CT on day 3 (day 3 of admission) shows extensive and diffuse GGOs with patchy consolidation. **(C,D)** Chest CT on day 7 shows diffuse consolidations worsening from GGOs with air bronchogram in both the lungs. CT, computed tomography; GGOs, ground-glass opacities.

**FIGURE 2:**
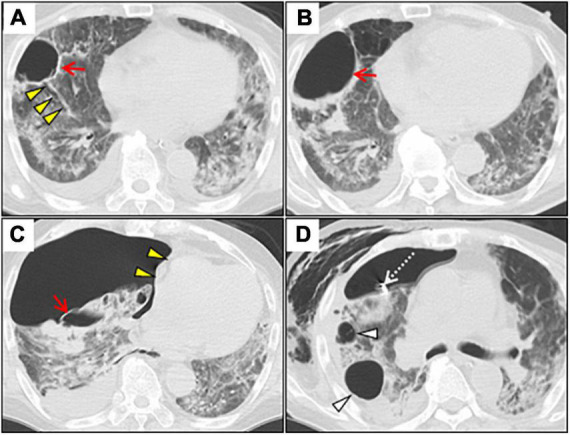
Serial chest CT images after induction of the invasive mechanical ventilation. **(A)** Axial chest CT on day 22 shows a lung cyst formation (red arrow) at the right S8 segment. Note the segmental bronchus connecting to the lung cyst (yellow arrowheads). **(B)** Chest CT on day 41 shows the gradually expanded cyst with air-fluid level, and wall thickening secondary to lung suppuration (red arrow). **(C)** Chest CT on day 43 shows a huge right-sided pneumothorax with mediastinal shift (yellow arrowheads). Note the collapsed cyst in the right segment 8 (red arrow). **(D)** Chest CT on day 51 shows residual air leaks after chest tube drainage (white dotted arrow), extending massive subcutaneous emphysema, and further enlargement of other lung cysts (white arrowheads). CT, computed tomography.

**FIGURE 3:**
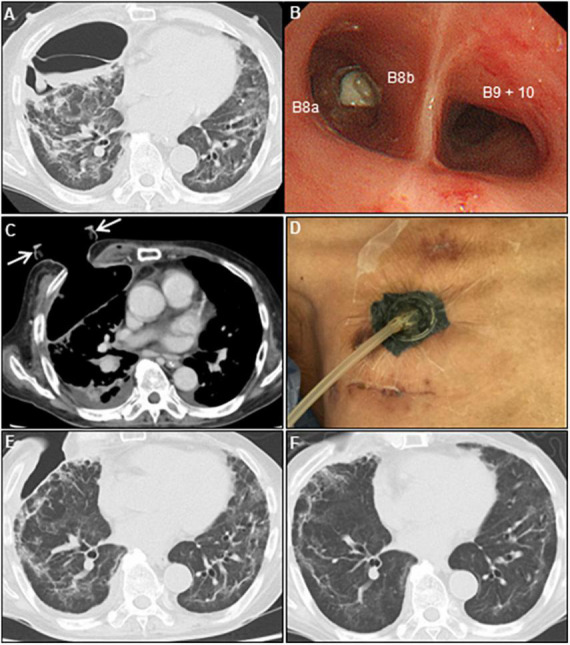
Multidisciplinary approach for persistent air leaks. **(A)** Chest CT on day 70 shows empyema and dense pleural thickening with air-fluid level. **(B)** Bronchoscope shows an endobronchial valve deployment inserted into the right B8b segment. **(C)** Post-minimally invasive OWT using a wound retractor (white arrows). **(D)** VAC system. Chest CT on day 97 **(E)** and 143 **(F)** show re-expansion of the collapsed lung parenchyma, and a repair of the chest wall after VAC therapy. CT, computed tomography; OWT, open-window thoracostomy; VAC, vacuum-assisted closure.

**FIGURE 4:**
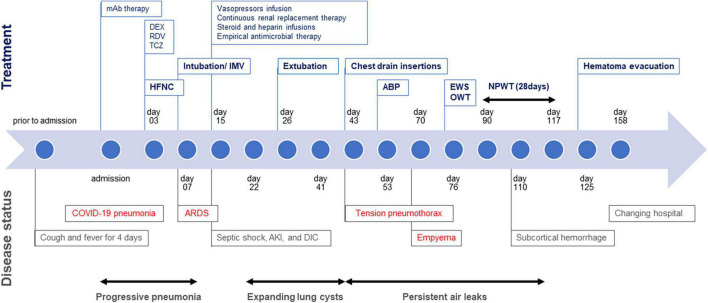
Timeline of case presentation.

A 73-year-old man was admitted to our hospital for general fatigue, presenting with symptoms of productive cough and fever for 4 days. He was a former smoker who had smoked 20 cigarettes a day for 20 years but had no pre-existing lung disease. His vital signs were as follows: blood pressure, 115/80 mmHg; heart rate, 110 beats/min; blood temperature, 36.2°C; respiratory rate, 24 breaths/min; and oxygen saturation, 90% on ambient air. Hematological examination revealed the following: white blood cell count, 2,600 cells/μL; differential count, 65.5% neutrophils, and elevated levels of C-reactive protein, 5.13 mg/dL (normal < 0.14 mg/dL); D-dimer, 10.1 μg/mL (normal < 1.0 μg/mL); LDH, 384 U/L (normal range, 124–222 U/L); and serum ferritin, 1,776 ng/mL (normal range, 20–200 ng/mL). SARS-CoV-2 infection was confirmed through reverse-transcriptase polymerase chain reaction (RT-PCR). Chest computed tomography (CT) on admission revealed patchy ground-glass opacities in both peripheral lungs, indicative of interstitial pneumonia (Figure 1A). A monoclonal antibody therapy directed against the spike protein of SARS-CoV-2 (casirivimab–imdevimab, 600/600 mg as a single intravenous dose) was initiated for moderate COVID-19 pneumonia; however, it was ineffective. On day 3, the patient continued to worsen clinically with progressive ground-glass opacities observed on the follow-up chest CT (Figure 1B). Thus, oxygen was administered with a high-flow nasal cannula (HFNC) at 40 L/min, with FiO_2_ titrated for oxygenation. In addition, we used a combination of oral dexamethasone (6 mg daily for 10 day) and IV remdesivir (200 mg, followed by 100 mg daily for 5 day), together with tocilizumab infusion (480 mg daily for 1 day). The chest CT on day 7 of admission revealed extensive ground-glass opacities, and diffuse consolidation with air bronchogram showing anteroposterior gradient in both the lungs, consistent with that of ARDS (Figures 1C,D). The patient developed severe hypoxemia of SpO_2_ 80%, despite HFNC oxygen therapy (FiO_2_ 1.0, 40 L/min), requiring intubation for respiratory insufficiency and IMV in the intensive care unit. The PaO_2_/FiO_2_ ratio was 120, suggestive of moderate ARDS. The IMV in the prone position was applied at a tidal volume of 6.6 mL/kg, positive end-expiratory pressure of 15 cm H_2_O, plateau pressure of 14 cm H_2_O, and respiratory frequency of 28/min. On day 15, a catheter-related bloodstream infection caused by *Enterobacter aerogenes* led to bacterial septic shock, consequent acute kidney injury and disseminated intravascular coagulation, requiring vasopressors, continuous renal replacement therapy, steroid infusion (Solu-Medrol 40 mg, daily for 16 day), and heparin infusion for 7 day. In addition, broad-spectrum antimicrobial treatment with meropenem (1 g/day IV for 10 day) was initiated, followed by antimicrobial de-escalation based on antimicrobial susceptibility test results (ceftriaxone, 4 g/day IV for 12 day). The chest CT on day 22 of admission revealed several lung cysts related to diffuse alveolar damage, predominantly on the right lung. Note the air-filled cystic lesion communicating to the segmental bronchus, was suspicious of a bronchopleural fistula (BPF) (Figure 2A). Follow-up RT-PCR confirmed SARS-CoV-2 negativity. On day 26, since the patient’s clinical status gradually improved, he was weaned off the IMV and extubated. The patient’s clinical condition remained stable thereafter; however, consecutive chest CT scans revealed progressive increase in size and number of lung cysts with a tendency to fuse with each other (Figure 2B). Two days later, the patient presented with dyspnea and severe chest pain. His vital signs were as follows: blood pressure, 90/70 mmHg; heart rate, 124 beats/min; respiratory rate, 38 breaths/min; and oxygen saturation, 83% on ambient air. The breath sounds were significantly diminished on the right side. Chest CT revealed a large right pneumothorax due to collapsed cysts with mediastinal shift, strongly suggestive of tension pneumothorax (Figure 2C). Air leaks had persisted despite two consecutive 20-Fr chest drain insertions (Figure 2D). On day 53, autologous blood pleurodesis (ABP) procedure was performed (100 mL, twice), but PALs were still observed. Moreover, collected material from chest cavity drainage tube was purulent, and CT findings on day 70 of admission were consistent with those of empyema (Figure 3A). On day 76, we attempted to facilitate healing of the PALs by inserting an Endobronchial Watanabe Spigot (EWS), a type of silicone bronchial blocker. Leak isolation performed *via* sequential balloon occlusion of the segmental bronchus using a bronchoscope revealed that the main source of the PALs was located in the right B8b segment, which was confirmed by an immediate reduction in air leaks on deploying a medium-sized EWS (Novatech, La Ciotat, France), and the procedure was completed (Figure 3B). Although air leaks recurred after an hour, bronchoscopy did not show any displacement of the implanted EWS, suggesting that the PALs were presumably due to myriad alveolopleural fistulas (APFs). Subsequent thoracoscopy revealed that the empyema cavity was too narrow for thoracoscopic manipulation. Therefore, minimally invasive open-window thoracostomy (OWT) using a wound edge protector was performed to eliminate PALs (Figure 3C and Supplementary Figure 1A). The incision length was 7 cm and surgical time was 105 min. Nine days after a dressing change, we clinically confirmed the cessation of air leaks. On day 90, negative-pressure wound therapy (NPWT) with a vacuum-assisted closure (VAC) device (KCI Medical Products, Winborne, Dorset, United Kingdom) was performed (Figure 3D). The pleural cavity was filled with GranuFoam (VAC Granufoam; KCI Medical, San Antonio, TX, United States), and covered with semipermeable films. Continuous suction was initially started at a negative pressure of 50 mmHg, and then maintained at a maximum negative pressure of 125 mmHg, alongside careful monitoring of the lung tissue damage. The dressings were changed twice per week. The patient well-tolerated these serial procedures, and experienced relief from dyspnea. NPWT for 28 days allowed re-expansion of the collapsed lung and enhanced wound granulation, resulting in closure of the thoracic cavity without the need for muscular flaps (Figures 3E,F and Supplementary Figures 1B-E). The postoperative course was uneventful. However, on day 110, the patient developed an extrapulmonary complication of a subcortical hemorrhage of the right parietal lobe, for which endoscopic hematoma evacuation was performed on day 125. Eventually, the patient was transferred to another hospital for further rehabilitation on day 158. At the 1-year follow-up, no recurrence of pneumothorax was observed. We present a timeline of the case in Figure 4.

## Discussion

The association between COVID-19 pneumonia and pneumothorax development has received increasing attention in the recent years. Previous retrospective and observational studies have shown that the incidence of pneumothorax is 1% in patients with COVID-19 pneumonia who need hospitalization, 2% in those who need intensive care treatment, and 5.9–15% in those receiving IMV (3–5). In a recent retrospective review examining 1,595 patients with COVID-19, pneumothorax occurred in 7% of patients, among whom IMV-related pneumothorax was diagnosed in 80% (1). Another retrospective study that examined 601 patients with COVID-19 pneumonia requiring IMV also supported the above epidemiological findings (4). Among patients requiring IMV, the frequency of barotrauma in the group with COVID-19 pneumonia was significantly higher than in the non-COVID-19 group (15 vs. 0.5%, *p* < 0.001), and in those with ARDS prior to the COVID-19 pandemic (15 vs. 10%, *p* < 0.001). Interestingly, pneumothorax occurs spontaneously in patients with COVID-19 pneumonia even in the absence of pre-existing lung disease or the need for IMV (6). Approximately 20% of patients with COVID-19 develop ARDS, which requires IMV (1). Given the high incidence rate of pneumothorax complicated by COVID-19-related ARDS, early detection and management of COVID-19-related pneumothorax is essential. While most cases of COVID-19-related pneumothorax resolve spontaneously or require chest tube drainage (1), some cases of refractory pneumothorax with PALs, secondary to COVID-19, eventually required thoracic surgery (7–9). However, COVID-19-related PALs pose diagnostic and therapeutic challenges.

Here, we described a refractory case of delayed tension pneumothorax in a patient with COVID-19-related PALs, that developed after IMV treatment for ARDS. This case provides the following two instructive clinical lessons.

Firstly, OWT-VAC therapy helped successfully control thoracic empyema with COVID-19-related PALs in our case.

A retrospective single-center study reported the details of the management and outcomes of COVID-19 complicated by pneumothorax (1). Patients having COVID-19 combined with pneumothorax were significantly associated with higher rates of in-hospital mortality than those without pneumothorax (58 vs. 13%, *p* < 0.001). Most patients having COVID-19 combined with pneumothorax (78%) required chest tube thoracostomy drainage for a median of 15 days (range, 2–86 days) with a median of one chest tube (range, 1–5 tubes). Large-bore chest tubes (≥ 20 F) were recommended over small-bore chest tubes (≤ 14 F) due to fewer tube-related complications. Approximately 5% of patients with pneumothorax ultimately required surgical intervention for PALs following tube thoracostomy drainage for a median of 47 days. PAL, defined as an air leak lasting for more than 5 days, can be caused by APF, BPF, or both. Although no solid guidelines exist for the management of COVID-19-related PALs, varied approaches have been documented in limited case reports and series. The surgical intervention techniques can be classified into two types depending on whether air leaks are identified. In cases of COVID-19 combined with refractory pneumothorax where air leaks can be identified anatomically, successful salvage lobectomy, surgical stapling, surgical resection of pneumatoceles, and thoracoscopic resection of blebs have been successfully performed to control PALs (1, 7–9). For patients with contraindications for surgery (advanced cancer, hemodynamic instability, severe hypoxemia, or very poor performance status), less invasive bronchoscopic interventions, such as use of endotracheal valves to seal BPFs or APFs, are indicated (10). Besides, a unique approach using endobronchial stents combined with occlusive materials for BPF closure has been reported. A combination of EWS with n-butyl-2-cyanoacrylate was successfully used to treat COVID-19-related BPFs in both elderly patients with a poor general condition complicated by multiple respiratory infections and middle-aged patients with alcoholic liver disease presenting with respiratory failure (11). In cases of COVID-19 with refractory pneumothorax where anatomical identification of air leaks is not possible, ABP is a non-surgical alternative to control COVID-19-related PALs. ABP is reportedly effective for the treatment of persistent pneumothorax with PALs in elderly patients with COVID-19 at high risk for surgery and anesthesia (12). ABP is a preferred, safe, and simple procedure for controlling PALs, with an overall success rate of approximately 92% (13). The proposed mode of action includes direct sealing of air leaks and induction of pleural inflammation, resulting in subsequent pleurodesis. However, serious complications, including tension pneumothorax caused by chest tube obstruction or empyema, may occur in < 10% of the cases (14).

In our case, adequate chest tube drainage and ABP failed to control COVID-19-related PALs, which were complicated by empyema, presumably due to procedure-related contamination or prolonged chest tube placement. Considering that the PALs persisted after endobronchial blockade in this case, the presence of residual APFs was strongly suspected. Therefore, we switched to OWT-VAC therapy, which is an ideal treatment option for empyema, eventually leading to a successful control of PALs. While conventional OWT, being minimally invasive, and allowing the direct drainage of empyema through the chest wall, effectively resolves the infections, the procedure requires resection of the ribs and intercostal muscles to permit repeated drainage and dressing of the cavity (15). Therefore, a delay in thoracostomy closure remains a concern, and a few cases warrant additional surgery. However, when combined with a VAC device, NPWT can facilitate drainage of the empyema and thoracic cavity closure, thereby shortening the length of hospital stay. NPWT is preferred over conventional therapies, owing to its advantage of faster wound healing. The following potentially beneficial effects have been considered (16): (1) a decrease in bacterial colonization of the affected tissue owing to increased clearance of infections and waste products; (2) increased circulation and oxygenation in damaged tissues owing to enhanced rapid angiogenesis; (3) reduction in interstitial edema; and (4) promotion of wound granulation, thus facilitating flap survival. Recently, NPWT has been extended to thoracic surgery. A cohort study that examined 19 patients with recurrent empyema revealed that NPWT more effectively reduced the empyema cavity, with the concurrent re-expansion of the residual lung tissue, leading to an early cure (17): The average duration of the OWT for patients undergoing VAC treatment (*n* = 11) was 39 ± 17 days versus 933 ± 1422 days for those not receiving VAC treatment (*n* = 8). Theoretically, NPWT carries the risk of aggravating BPFs and causing excessive negative-pressure-induced organ damage through the fistula, and hence, should be avoided. However, in patients with small-sized BPFs of ≤ 1 mm, NPWT is considered safe and effective for both empyema and BPF closure, under a negative pressure of 125 mmHg or less (18), by maximizing blood flow and not causing tissue damage, as proven in animal studies (19). Similarly, NPWT was safely performed in our patient with PALs caused by APFs. In addition, minimally invasive OWT using a wound retractor (XS size; Applied Medical, Rancho Santa Margarita, CA, United States) allowed minimal stoma and rib resection, maintained wound patency, and permitted daily dressing changes (20). Moreover, the combination with the VAC device induced re-expansion of the residual lung tissue and contributed to the closure of the APFs by presumably contacting the chest wall and adjacent lung lobes, thus controlling PALs. Therefore, this case highlights the potential of OWT-VAC therapy as a promising therapeutic alternative to control COVID-19-related PALs, refractory to multiple surgical interventions.

This approach has the following four possible limitations. First, as described above, NPWT is originally not indicated for treating BPFs due to the risk of negative pressure-related organ damage through the fistula (17). However, several successful cases of BPF with NPWT have been reported: NPWT performed at a negative pressure of 75–125 mmHg was effective for a 1-mm BPF but not for an 8-mm BPF (18). Therefore, in cases of large BPFs, the fistula should be controlled by either bronchoscopic or surgical interventions before NPWT. Second, for patients with poor performance status and long-term hospitalization, OWT with rib resection may have further reduced the activities of daily living due to pain. Third, there may be a residual risk of uncontrollable APFs even after OWT-VAC therapy as it does not involve radical closure of APFs. A final limitation is that additional invasive thoracoplasty may be necessary to reduce the thoracic cavity volume in case of residual free space in the thoracic cavity even after the thoracic empyema has healed. Therefore, further investigation of the efficacy of OWT-VAC therapy in refractory pneumothorax with PALs is warranted.

Secondly, serial CT monitoring facilitated the detection of pneumothorax secondary to COVID-19-related ARDS and for understanding its pathogenesis in our case.

The pathogenesis of COVID-19-related pneumothorax remains poorly understood and is considered multifactorial. It involves barotrauma, a type of ventilator-induced lung injury (21), and radiological cystic features of the lungs, which may be attributable to adverse lung processes caused by severe SARS-CoV-2 infection (22). However, the latter remains controversial due to the spontaneous resolution of cystic features in some cases while pneumothorax may occur, without accompanying cystic changes (12, 23). In addition, a patient’s self-inflicted lung injury (P-SILI) or steroids can influence pneumothorax development (24, 25). Considering that the lung cysts that developed during IMV rapidly expanded after extubation and consequently ruptured, it is highly likely that lung cysts with barotrauma-induced APFs maintained their sizes during IMV, under a lung-protective strategy. However, after IMV, they acutely progressed to rupture owing to P-SILI, which increased the volume and negative intrathoracic pressure during spontaneous single-lung ventilation, resulting in a delayed pneumothorax in this case. In addition, prolonged steroid treatment may have contributed to the lung fragility, rendering them prone to cystic degeneration. A systematic review of air leaks in COVID-19 patients showed that the average time from symptom onset to diagnosis of pneumothorax was 11.63 days (range, 1–30 days), except for a single patient (26) who developed pneumothorax after 56 days. Furthermore, several cases of recently resolved COVID-19 pneumonia have been reported for readmission with tension pneumothorax, approximately 3 weeks after symptom onset (27, 28). Our patient developed delayed tension pneumothorax 47 days after symptom onset, and 15 days after IMV withdrawal, which is the second latest manifestation of COVID-19, and rare to the best of our knowledge. Therefore, this case illustrates the significance of considering tension pneumothorax in patients showing rapid hemodynamic instability despite the resolution of COVID-19-related ARDS.

In conclusion, we reported a case of COVID-19-related PALs with delayed tension pneumothorax after IMV for ARDS. The PALs caused by APFs were refractory to multiple surgical interventions and complicated by empyema, which was eventually cured with minimally invasive OWT-VAC treatment. To the best of our knowledge, this is the first case report to describe this unique technique. Further evidence is warranted to validate OWT-VAC therapy for empyema with COVID-19-related PALs. Clinicians should be fully aware of the possibility of serious sequelae of pneumothorax in COVID-19 patients, even after associated ARDS resolution. Close CT monitoring in severe cases of COVID-19 pneumonia can be beneficial, and lung cysts should be monitored carefully for its susceptibility to secondary pneumothorax.

## Data availability statement

The original contributions presented in this study are included in the article/Supplementary material, further inquiries can be directed to the corresponding author.

## Ethics statement

A written consent was obtained from the patient. Furthermore, the authorization for waiver of consent was approved by the Institutional Review Board (IRB) of Narita-Tomisato Tokushukai Hospital with the permission of the director, HO. All images in the current case are entirely unidentifiable, and patient anonymity is completely preserved. The head of the IRB was responsible for anonymizing the patient.

## Author contributions

HY was responsible for the clinical study design and conceptualization of the study. KK, TN, TM, TS, HM, and HO were involved in the acquisition of clinical data. HY, TN, and JI analyzed and interpreted the data. HY and JI drafted the manuscript. All authors discussed, read, and approved the submission of this manuscript for publication.

## Abbreviations

ABP: autologous blood pleurodesis
AKI: acute kidney injury
APF: alveolopleural fistula
ARDS: acute respiratory distress syndrome
BPF: bronchopleural fistula
COVID-19: coronavirus infections 2019
CT: computed tomography
DEX: dexamethasone
DIC: disseminated intravascular coagulation
EWS: endobronchial Watanabe spigot
HFNC: high-flow nasal cannula
IMV: invasive mechanical ventilation
mAb: monoclonal antibody
NWPT: negative-pressure wound therapy
OWT: open-window thoracostomy
PALs: persistent air leaks
P-SILI: patient self-inflicted lung injury
RDV: remdesivir
RT-PCR: reverse-transcriptase-polymerase chain reaction
SARS-CoV-2: severe acute respiratory syndrome coronavirus type 2
TCZ: tocilizumab
VAC: vacuum-assisted closure.
