# Supplementary material for: Negative-pressure wound therapy to treat thoracic empyema with COVID-19-related persistent air leaks: A case report
Source: Front Med (Lausanne). 2022 Aug 11;9:970239. doi: 10.3389/fmed.2022.970239 (PMC9402970; doi:10.3389/fmed.2022.970239)
Supplement: Supplementary file 1 [file Table_1.DOCX]

Supplementary Material

# Supplementary Data

**Supplementary Figure 1.** Wound healing process

(A) Post-minimally invasive open-window thoracostomy with a wound retractor (POD0). (B) 3 days after the VAC therapy (POD17). (C) 10 days after the VAC therapy (POD24). (D) 28 days after the VAC therapy (POD42). (E) 53 days after the VAC therapy (POD67). POD, day after open-window thoracostomy; VAC, vacuum-assisted closure.
