# Supplementary figures and images for: Negative-pressure wound therapy to treat thoracic empyema with COVID-19-related persistent air leaks: A case report
Source: Front Med (Lausanne). 2022 Aug 11;9:970239. doi: 10.3389/fmed.2022.970239 (PMC9402970; doi:10.3389/fmed.2022.970239)

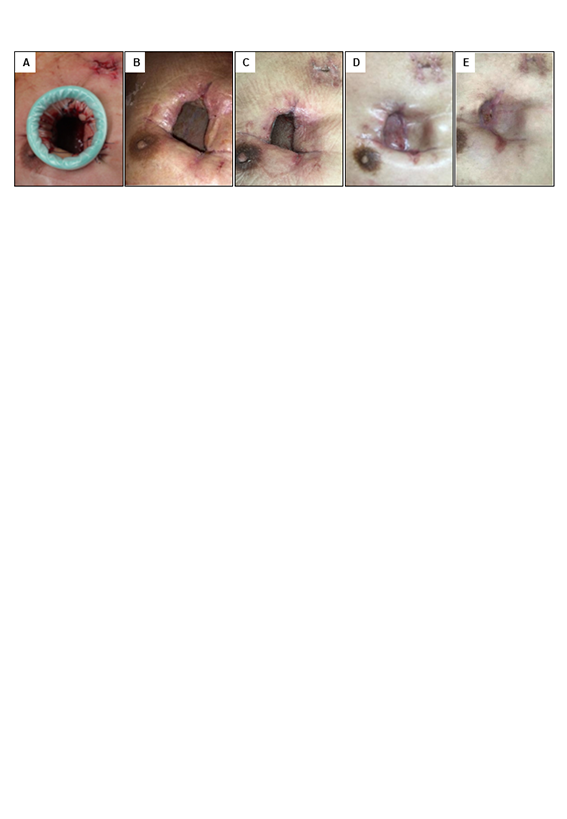

Supplement: Supplementary file 2 [file Image_1.TIF]
